# Reliability of home CPAP titration with different automatic CPAP devices

**DOI:** 10.1186/1465-9921-9-56

**Published:** 2008-07-24

**Authors:** Frédéric Sériès, Julie Plante, Yves Lacasse

**Affiliations:** 1 Unité de recherche en pneumologie, Centre de recherche de l'Hopital Laval, Institut Universitaire de cardiologie et de pneumologie de l'Université Laval, Quebec City, Canada

## Abstract

**Background:**

CPAP titration may be completed by automatic apparatus. However, differences in pressure behaviour could interfere with the reliability of pressure recommendations. Our objective was to compare pressure behaviour and effective pressure recommendations between three Automatic CPAP machines (Autoset Spirit, Remstar Auto, GK 420).

**Methods:**

Sixteen untreated obstructive sleep apnea patients were randomly allocated to one of the 3 tested machines for a one-week home titration trial in a crossover design with a 10 days washout period between trials.

**Results:**

The median pressure value was significantly lower with machine GK 420 (5.9 +/- 1.8 cm H_2_O) than with the other devices both after one night and one week of CPAP titration (7.4 +/- 1.3 and 6.6 +/- 1.9 cm H_2_O). The maximal pressure obtained over the one-week titration was significantly higher with Remstar Auto (12.6 +/- 2.4 cm H_2_O, Mean +/- SD) than with the two other ones (10.9 +/- 1.0 and 11.0 +/- 2.4 cm H_2_O). The variance in pressure recommendation significantly differed between the three machines after one night and between Autoset Spirit and the two other machines after 1 week.

**Conclusion:**

Pressure behaviour and pressure recommendation significantly differ between Auto CPAP machines both after one night and one week of home titration.

## Background

Obstructive sleep apnea hypopnea syndrome (OSAHS) is highly prevalent in the middle age active population [1,2]. The consequences of obstructive breathing disturbances on sleep structure and continuity, tissue oxygenation, hemodynamic variables and on the release of systemic inflammatory mediators can account for vigilance and quality of life impairments [3] as well as for the increase in morbidity and mortality [4-7]. Nasal continuous positive airway pressure (CPAP) represents a very effective treatment for OSAHS as demonstrated by the results of different randomized controlled [8-11] and non-randomized trials [12].

The effective pressure level (Peff) is the one that abolishes obstructive breathing disorders including inspiratory flow limitation and snoring in every sleep stage and body position [13]. It is usually determined during an in-laboratory titration sleep study with continuous acquisition of electrophysiologic variables, instantaneous respiratory flow, respiratory efforts and pulse oximetry. Automatic CPAP devices have been developed that continuously adapt the applied pressure level to the ventilatory profile [14]. These devices may be used in two different ways, one is to replace the in-laboratory titration sleep study and determine the Peff level in the patient's usual sleeping environment. These automatic titration procedures have been widely used in the literature [8,10,11] and can be used in clinical practice in the management of CPAP therapy [15,16]. Then when completing an automatic CPAP titration, Peff value usually corresponds to the 90^th ^or 95^th ^percentile of the cumulative night time pressure response. The other application of auto CPAP therapy is to simply replace conventional fixed CPAP therapy at home by a machine that should automatically modify the pressure setting accounting for intra night and night-to-night changes in Peff [17].

Different automatic CPAP devices are presently available that differ in the analyzed signals, in the definition of respiratory events, in the signal processing as well as in the algorithm of pressure response [18-24]. This results in significant differences in the positive pressure behaviour in response to bench-simulated [25,26] or naturally occurring sleep-induced breathing disturbances [27]. One should expect such specificity in the machine pressure response to influence the amount and duration of pressure changes and consecutively the pressure recommendation between different automatic CPAP machines. This has been found to be the case when comparing in-laboratory [28] as well as home [29] titration results with two Auto CPAP devices (Autoset, Resmed, Sydney Australia and Somnosmart, Weinmann, Hamburg, Germany) whose response algorithms are based on entirely different physiologic principles (i.e. correction of apnea/hypopnea and flow/time profile vs. maintenance of respiratory system impedance below awake values while asleep respectively). Nolan recently reported that pressure delivery significantly differs between machines using different algorithms of pressure response that are driven by respiratory flow analysis [30], but the impact of such differences on positive pressure setting cannot be drawn from this last study since it was conducted in patients previously treated with CPAP with no aim to establish and compare positive pressure recommendations.

The aims of the present study were to compare pressure behaviour and Peff recommendations between three different Automatic CPAP machines and evaluate if these parameters are influenced by titration duration.

## Methods

Sixteen newly diagnosed consecutive subjects participated in the study. The diagnosis of sleep apnea was made according to clinical symptoms and to the results of ambulatory (oxygen desaturation index > 15/h, n = 8) or in-laboratory sleep recordings (AHI > 10/h, n = 8). These nocturnal recordings were scored according to recommendations of the literature [31,32]. Patients selected CPAP as their treatment choice after discussion of other treatment alternatives. No patient had ever been treated for OSAHS. Their weight had to be stable over the last 2 months with no change in medication during this period. They were asked not to initiate a weight loss strategy before the end of the study. Patients should have normal nasal ventilation and no documented obstructive or restrictive lung disease, neuromuscular disorder or congestive heart failure. They should not be taking any CNS/respiratory depressant medication and alcohol consumption had to be less than 0.5 g alcohol/Kg per day. Patients whose diurnal somnolence represented an urgent indication for treatment according to the referring pneumologist were not eligible to participate. The ethical review board approved the protocol and subjects provided informed written consent.

### Study design

An experienced sleep technologist who also explained to patients the functioning of Auto CPAP devices made the cautious choice of the nasal mask. Patients were randomly allocated to one of the investigated Auto CPAP machines (GK 420 – Tyco Healthcare International, AutoSet Spirit – ResMed, Sydney Australia, Remstar Auto – Respironics, Murrysville, PA) for a one-week home titration trial in a crossover design with a 10 days washout period between trials. Each machine was used with default settings with lower and upper pressure bounds set to 4 and 16 cm H_2_O respectively. Anthropomorphic characteristics were measured at the beginning of each trial. At the end of each treatment session, information recorded by the Auto CPAP device was downloaded to determine adherence to treatment, to measure apnea + hypopnea index and leaks level, applied pressure range, median pressure value, and default values of the recommended Peff level (90^th ^percentile pressure levels for GK 420 and RemStar Auto and 95^th ^for AutoSet Spirit). These parameters were determined separately for the first night and the whole week of treatment.

### Statistical analysis

The sample size was determined according to the results of a pilot study conducted in 8 subjects to observe a 90% chance of showing a 2 cm H_2_O statistical difference in the recommended pressure level between the 3 tested machines with a two-sided significant level of 5%. The results obtained in these 8 subjects are not part of the present results. For the one night and one-week recording sessions, analyzed variables obtained from machines' reports were CPAP compliance, AHI, pressure characteristics (maximum, minimum, median and recommended effective pressure levels). Data recorded on "night 1" were analysed using a randomized block design (mixed model). Subjects were linked to the block effect (random effect) and machines (Autoset Spirit, GK-420 and RemStar Auto) were associated to the fixed effect. The Tukey's adjustment was used to perform *posteriori *multiple comparisons. The same statistical approach was used for measurement over the week recording. To compare night1 and one-week recordings, a second fixed factor was added to the previous statistical model with an interaction effect with machines. The univariate and multivariate normality assumption were verified with the Shapiro-Wilk and Mardia's tests respectively. The results were considered significant with p-values ≤ 0.05. The data were analyzed using the statistical package program SAS v 9.1.3 (SAS Institute Inc.).

## Results

The characteristics of participating patients are reported in Table 1. Symptoms, co-morbidities and anthropometric variables did not differ between patients whose diagnosis was confirmed with ambulatory or in-laboratory sleep recording. Anthropometric variables remained unchanged at the different visits. No change in mask was required within the different titration sessions or from one session to the other. No subject complained of any symptom of nasal obstruction during the course of the study. No data loss occurred except in one patient who repeatedly turned the machine on and off during the first night of the last treatment session with Remstar Auto. CPAP usage was similar between the three machines (table 2 and 3). The apnea + hypopnea index (AHI) remained greater than 10/h in 2/16 subjects during the first night titration with Remstar Auto and in 2 other subjects during the one week titration (one with Remstar Auto and GK 420). AHI values significantly differed between the 3 devices both after one night and one week of titration (Table 2 and 3). Leaks remained in the normal value range in each subject (less than 0.4 L/min with Autoset Spirit, less than the maximal computed leak level with GK-420 and without periods of "large leaks" indicated by the RemStar Auto). The maximal pressure reached did not differ between the three Auto CPAP devices for the first night but its variance was significantly higher with GK-420 (10.7) than Autoset Spirit and RemStar Auto (1.64 and 5.47 respectively, p < 0.05). The maximal pressure obtained over the one-week titration was significantly higher with the RemStar Auto than with the two other ones (Table 2 and 3). Furthermore, the maximal pressure variance of the one-week recording was significantly higher for RemStar Auto and GK-420 (4.31 and 5.71 respectively) than Autoset Spirit (1.22) (p < 0.05). The minimal pressure level reached 4 cm H_2_O with the three devices for each titration duration. The median pressure value was significantly lower with GK-420 than with the other devices both after one night and one week of CPAP titration (Table 2 and 3). Furthermore, the median pressure variance measured at one week was borderline significant between the 3 machines (Autoset Spirit: 1.86, RemStar Auto: 3.31, GK-420: 3.01, 0.05 < p <0.1). The difference in median pressure values measured overtime was significantly less for GK-420 than for the two other machines (p = 0.003). The intra-night pressure variability was estimated by the difference between the effective and median pressure values. This variability was significantly lower with RemStar Auto than Autoset Spirit following the first night and with REMStar than with the two other devices after one-week titration (Table 2 and 3).

**Table 1:** Characteristics of participating subjects.

Age (y)	49 (38 – 65)
Sex	14 M/2F
BMI (Kg/m^2^)	30.8 ± 4.5
ESS	12.3 ± 1.1
Co morbidities	HBP (n = 6), CAD (n = 4), diabetes (n = 4), gout (n = 1), GERD (n = 1)
Medications	Beta blockers (n = 4), ACE (n = 2), AR blockers (n = 2), diuretics (n = 2), nitrates (n = 2), Allopurinol (n = 1).
RDI (n/h)	38. 5 ± 20.0

ESS: Epworth Sleepiness score, RDI: respiratory disturbances index. HBP: high blood pressure, CAD: Coronary artery disease, GERD: Gastro-oesophageal Reflux Disease, Mean (range) or SD

**Table 2:** Results of the titration studies according to each Auto CPAP device during the first titration night.

Auto CPAP device	Autoset Spirit	Remstar Auto	GK 420
CPAP usage (h)	6.2 ± 1.9 ^a^	4.6 ± 2.4 ^a^	5.9 ± 1.6 ^a^
AHI (n/h)	8.0 ± 5.6 ^a^	4.2 ± 3.1 ^b^	2.9 ± 3.0 ^c^
Max pressure (cm H_2_O)	11.1 ± 1.4 ^a^	10.9 ± 2.4 ^a^	11.5 ± 3.3 ^a^
Median pressure (cm H_2_O)	7.4 ± 1.3 ^a^	7.1 ± 1.8 ^a^	5.7 ± 1.8 ^b^
Recommended pressure level (cm H_2_O)	9.9 ± 1.2 ^a^	9.6 ± 2.0 ^a^	9.3 ± 2.9 ^a^
Pressure variability	2.6 ± 0.9 ^a^	1.8 ± 0.9 ^b^	3.0 ± 1.9 ^ab^

For a given variable (row), columns assigned with different letters denote statistically significant difference. Conversely, for a given variable, columns that are sharing a same given letter are not significantly different. Mean ± SD.

**Table 3:** Results of the titration studies according to each Auto CPAP device during the whole week titration.

Auto CPAP device	Autoset Spirit	Remstar Auto	GK 420
CPAP usage (h)	5.6 ± 1.9 ^a^	4.6 ± 1.7 ^a^	5.0 ± 2.3 ^a^
AHI (n/h)	7.1 ± 4.1 ^a^	4.7 ± 3.4 ^b^	3.1 ± 3.1 ^c^
Max pressure (cm H_2_O)	10.9 ± 1.0 ^a^	12.6 ± 2.4 ^b^	11.0 ± 2.4 ^a^
Median pressure (cm H_2_O)	7.4 ± 1.3 ^a^	6.6 ± 1.9 ^a^	5.9 ± 1.8 ^b^
Recommended pressure level (cm H_2_O)	9.8 ± 1.1 ^a^	9.0 ± 2.0 ^a^	9.2 ± 2.7 ^a^
Pressure variability	2.6 ± 0.5 ^a^	1.8 ± 1.0 ^b^	2.9 ± 1.6 ^a^

Significant differences between columns are indicated by the use of different letters as described in table 2.

**Figure 1:**
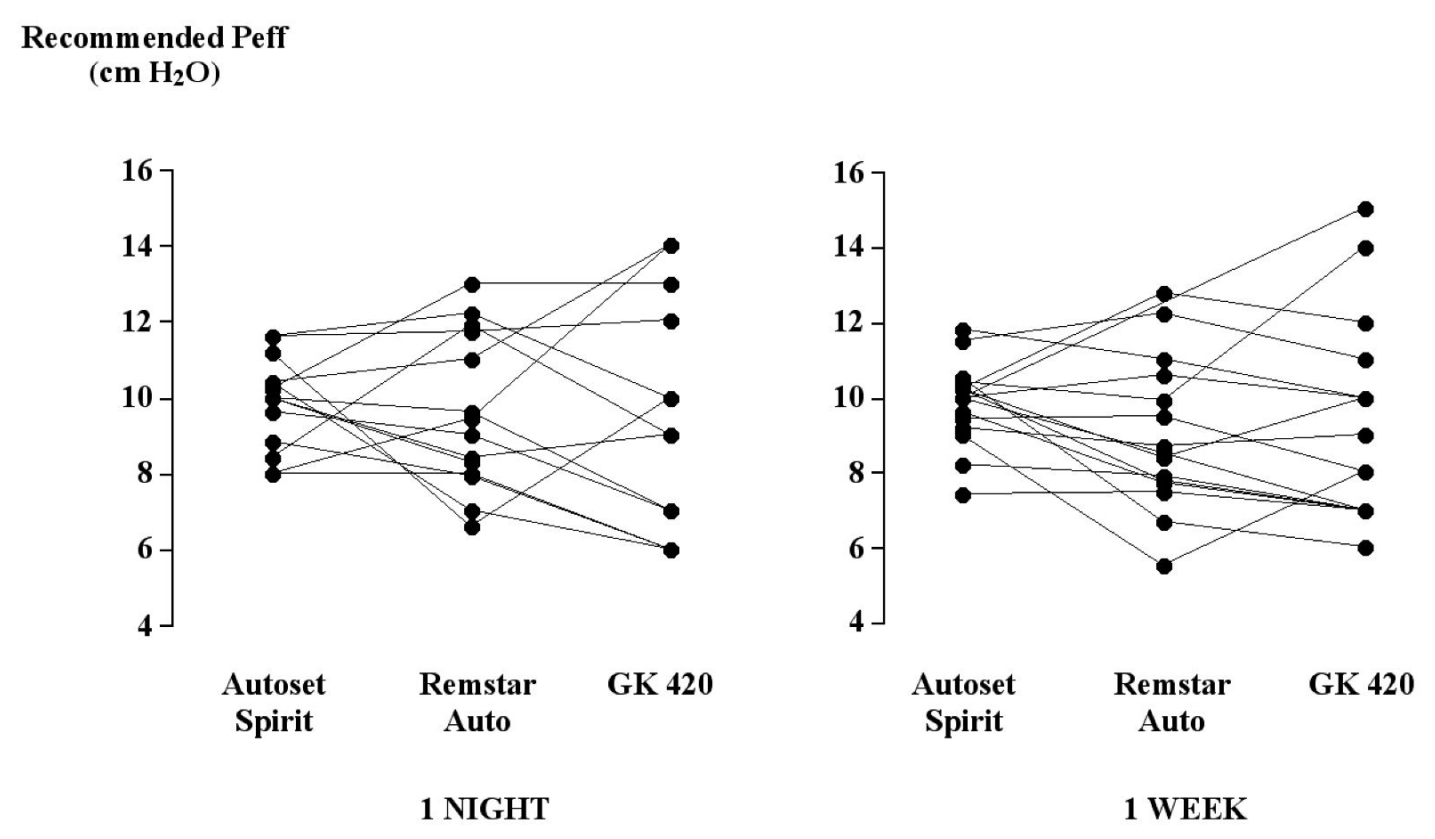
Individual values of the recommended effective pressure (Peff) level obtained after one night and one week of automatic CPAP titration with the three tested apparatus.

**Figure 2:**
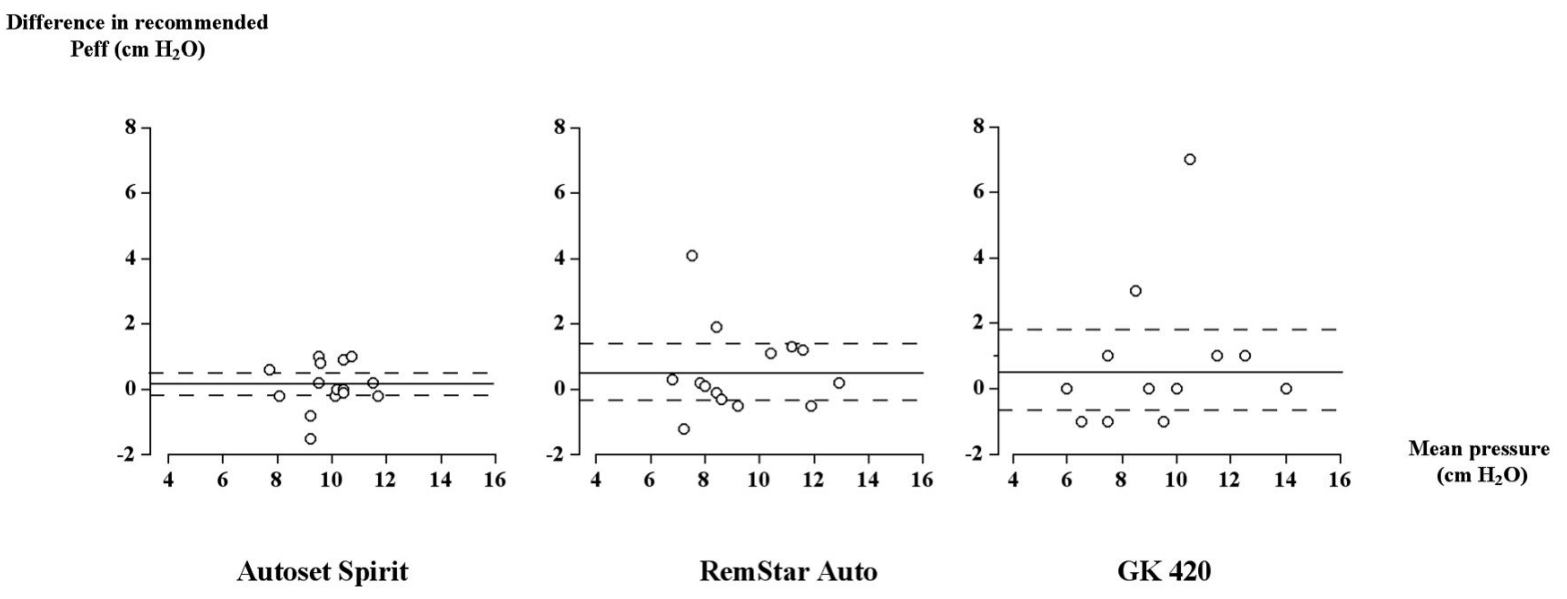
**Bland and Altman representation of the recommended effective pressure (Peff) level obtained after one night minus Peff at one week of automatic CPAP titration against the corresponding mean Peff value for each tested apparatus.** (Mean ± 95% CI).

The mean values of recommended pressures obtained did not significantly differ between the three machines both after one night and one week of titration (Table 2 and 3). However, important variations in machine-to-machine Peff recommendations were seen (Figure 1). Significant differences were observed in the variance of Peff recommendation between the three machines after one night (Autoset Spirit: 1.21, RemStar Auto: 3.96, GK-420: 11.37) (p < 0.001), this difference being significant between machine Autoset Spirit (1.42) and RemStar Auto and GK-420 (3.45 and 7.35 respectively) after 1 week (p < 0.05). No significant difference was found in the mean Peff values obtained after one night and one week of auto titration. However, this stability in Peff values was seen at the expense of important increases and decreases in pressure setting recommendations overtime (Figure 2), but without systematic bias with increasing recommended pressure setting. The variance of the changes in Peff overtime significantly differed between the three machines (Autoset Spirit: 0.4, RemStar Auto: 1.81, GK-420: 5.85) (p < 0.01). The machine-to-machine variability in pressure setting recommendations significantly exceeded the time-dependent variability of this measurement.

No significant sequence and period effects were found for any of the studied variables. The above-detailed results were not influenced by type of recording (ambulatory or in-lab polysomnography) used to ascertain the presence of sleep apnea.

## Discussion

Our results demonstrate that pressure behaviour significantly differs between Auto CPAP machines both after one night and one week of home titration. They also indicate that the duration of the titration procedure does not contribute to dampen the difference in the titration results between the different machines. Furthermore, on an individual basis, pressure recommendations were found to be highly variable with time. This implies that the results of an auto titration procedure completed with one of the investigated device cannot be considered as similar to those coming from a same titration protocol completed with another auto titrating machine whatever the duration of the titration protocol. It also indicates that completing the auto titration procedure for a week does not provide additional information to a one-night recording,

We recognize that this study has some weaknesses such as patient selection criteria and lack of in-lab evaluation of positive pressure requirements. As previously stated, patients whose diagnosis was confirmed with an ambulatory/in-lab sleep recording had identical clinical complaints, anthropometric characteristics and co-morbid conditions. We acknowledge that the obstructive nature of sleep-disordered breathing cannot be firmly ascertained by the analysis of oximetry recordings [33]. However, the absence of heart or respiratory failure in the participating subjects, the fact that none of them was taking any CNS/respiratory depressant medication and the ability of positive pressure therapy to abolish nocturnal breathing disorders strongly support the obstructive nature of breathing disturbances as supported by recent data published by Mulgrew et al [34]. For these reasons, we are confident that our results are not biased by the fact that different types of sleep recording ascertained the presence of OSA. A second possible drawback relates to the absence of in-lab assessment of positive pressure needs. The aims of the present study were to compare the pressure behaviour of different automatic CPAP devices used in a home CPAP titration setting. In this context, it is not possible to ascertain that the effective pressure level remained unchanged from one automatic CPAP session to the other. However, the fact that the machine-to-machine variability in Peff recommendation exceeded the time-dependent variability of this variable rules out a potential effect of day-to-day (or week-to-week) variability in positive pressure needs in the difference in pressure recommendations between the three machines. We are also not able to compare pressure settings coming from automatic and manual titration (using respectively home and in-lab titration procedures) for each machine. Such comparison would require the completion of an in-laboratory titration following the first night and the one-week automatic titration procedures for each machine. However, it is important to remember that the tested machines were able to normalize breathing abnormalities (or near so) in each circumstance. Considering that identification of breathing disorders and pressure behaviour are intimately linked, it would be interesting to have access to machines' raw data of flow/pressure recordings and also to complete simultaneous conventional analysis of respiratory disturbances using sleep and ventilatory recordings during auto CPAP titration to establish the nature of breathing disorders that drive pressure changes.

Pressure behaviour has been found to significantly differ among Automatic CPAP machines in response to predefined breathing disturbances in different bench studies [25,26]. Our study is the first to illustrate the importance of such behaviour on pressure setting recommendations when these machines are used to determine an effective pressure level at home. Recently West et al found no difference in CPAP usage, nor in the improvement in daytime vigilance and quality of life between patients being treated with fixed CPAP (titration being completed either with a dedicated algorithm or with one week auto-titration procedure) or automatic CPAP [35]. However, the effective pressure levels obtained in each group were remarkably close, probably due to the homogeneity of the study sample. In the present study, the range of the difference in recommended pressures was wide (-3.5 to 3.4 cm H_2_O between Autoset and REMStar auto, -6.0 to 4.4 cm H_2_O between Autoset and GK 420 and -2.9 to 3.4 cm H_2_O between REMStar auto and GK 420). Considering that a difference between pressure setting and effective pressure level ≥ 1 cm H_2_O may interfere with treatment efficiency [13,36], the differences in pressure behaviour that we observed may have important clinical significance when the recommended pressures are considered as the ones to be set for conventional treatment with a fixed CPAP. It is reasonable to assume that such differences in recommended pressure settings may be associated with persistence of residual obstructive breathing disorders and incomplete relief of nocturnal and diurnal complaints. The present results are particularly important when it has been shown that there is no advantage of auto-CPAP therapy compared to fixed CPAP calibrated according to an automatic CPAP home trial [35].

The variability in Peff that we observed can theoretically be attributed to differences in sleeping conditions and/or in the different components of algorithm of pressure response. There is no reason to believe that the factors known to influence pressure setting such as body, head and neck position and night to night variability should differ from one home titration session to the other. Therefore, the fact that the machine-to-machine variability in pressure setting recommendations significantly exceeded the time-dependent variability of this measurement implies that the observed variability in pressure setting relates to differences in the machines pressure responses. Auto CPAP machines may use different signal processing of the flow signal and different definitions of respiratory events (hypopnea, flow limitation), may respond differently to these events (blocking in pressure rise in the absence of improvement in flow profile, rate and amount of pressure rise), may apply different plateauing duration before stepping down the pressure level, and may respect different rate and amount of pressure decrease. For example, the response to apneic events leads to a maximal increase in pressure of 2 cm H_2_O with the Autoset Spirit but to a 1 cm H_2_O pressure increament/15s with the REMstar Auto. In response to flow limitation, the respective pressure rise is 0.3 cm H_2_O/breath and 0.5 cm H_2_O/min for these two devices. The duration of pressure plateauing varies between 5 breaths and 5 minutes between the three tested machines and the range of pressure decrease goes from 0.2 cm H_2_O/breath to 0.5 cm H_2_O/minute. As a consequence of the differences in the machines algorithm of pressure response, the variability of the applied pressure levels was found to significantly differ between the tested devices (recommended pressure/median pressure ratio) despite similar mean recommended pressure values. The present findings support the importance of machine-to-machine differences in pressure behaviour as a contributor to the heterogeneity between CPAP levels in trials comparing fixed and automatic CPAP therapy [37]. On the other hand, such result implies that clinical results obtained with one given automatic CPAP machine are specific to the tested apparatus and cannot be applied to other devices (i.e. to evaluate treatment benefits, to identify patients who would particularly benefit of such form of CPAP treatment). This opens the door to a possible specificity of the usefulness of a given automatic CPAP devices depending on the characteristics of sleep-induced disordered breathing and of the pressure response profile of this device. In such case, it is conceivable that patients requiring high pressure levels could benefit of using an auto CPAP machine different from patients who have positional or sleep-stages dependent breathing disturbances.

## Conclusion

There is an important variability in CPAP level recommendations between automatic CPAP machines used for home titration and that performing a prolonged titration procedure does not reduce machine-to-machine variability. Such differences may lead to important discrepancies in the effective pressure level of pressure and consequently alter CPAP compliance and/or efficiency. Considering the impact of CPAP therapy on sleep apnea-related morbidity and mortality and the potential benefits of automatic CPAP titration as a cost and time effective method to initiate an effective treatment, clinical studies are needed to better define the specificity and clinical adequacy of the different algorithm of pressure response in determining the effective pressure level during home titration with automatic CPAP devices. Such studies are particularly important to refine our ability to make a routinely adequate usage of these automatic machines.

## Competing interests

The authors declare that they have no competing interests.

## Authors' contributions

FS conceived of the study, elaborated its design and contributed to its coordination. JP and YL participated in the revision of the design of the study. All authors participated in and helped to draft the manuscript. All authors read and approved the final manuscript.

## References

[B1] Young T, Palta M, Dempsey J, Skatrud J, Weber S, Badr S (1993). The occurrence of sleep-disordered breathing among middle-aged adults. N Engl J Med.

[B2] Hiestand DM, Britz P, Goldman M, Phillips B (2006). Prevalence of symptoms and risk of sleep apnea in the US population: Results from the national sleep foundation sleep in America 2005 poll. Chest.

[B3] Vgontzas AN, Papanicolaou DA, Bixler EO, Hopper K, Lotsikas A, Lin HM, Kales A, Chrousos GP (2000). Sleep apnea and daytime sleepiness and fatigue: Relation to visceral obesity, insulin resistance, and hypercytokinemia. Journal of Clinical Endocrinology and Metabolism.

[B4] Grote L, Ploch T, Heitmann J (1999). Sleep-related breathing disorder is an independent risk factor for systemic hypertension. Am J Respir Crit Care Med.

[B5] Shahar E, Whitney CW, Redline S, Lee ET, Newman AB, Javier Nieto F, O'Connor GT, Boland LL, Schwartz JE, Samet JM (2001). Sleep-disordered breathing and cardiovascular disease: Cross-sectional results of the sleep heart health study. Am J Respir Crit Care Med.

[B6] Peppard P, Young T, Palta M, Skatrud J (2000). Prospective study of the association between sleep-disordered breathing and hypertension. N Engl J Med.

[B7] He J, Kryger MH, Zorick FJ, Conway W, Roth T (1988). Mortality and apnea index in obstructive sleep apnea. Experience in 385 male patients. Chest.

[B8] Senn O, Brack T, Matthews F, Russi EW, Bloch KE (2003). Randomized short-term trial of two auto-CPAP devices versus fixed continuous positive airway pressure for the treatment of sleep apnea. Am J Respir Crit Care Med.

[B9] Engleman HM, Kingshott RN, Wraith PK, Mackay TW, Deary IJ, Douglas NJ (1999). Randomized placebo-controlled crossover trial of continuous positive airway pressure for mild sleep Apnea/Hypopnea syndrome. Am J Respir Crit Care Med.

[B10] Jenkinson C, Davies DO, Mulins R, Stradling JR (1999). Comparison of therapeutic and subtherapeutic nasal continuous positive airway pressure for obstructive sleep apnoea: a randomized prospective parallel trial. Lancet.

[B11] Masa JF, Jimenez A, Duran J, Capote F, Monasterio C, Mayos M, Terán J, Hernández L, Barbé F, Maimó A, Rubio M, Montserrat JM (2004). Alternative methods of titrating continuous positive airway pressure: a large multicenter study. Am J Respir Crit Care Med.

[B12] Marin JM, Carrizo SJ, Vicente E, Agusti AG (2005). Long-term cardiovascular outcomes in men with obstructive sleep apnoea-hypopnoea with or without treatment with continuous positive airway pressure: an observational study. Lancet.

[B13] Meurice JC, Paquereau J, Denjean A, Patte F, Sériès F (1998). Influence of correction of flow limitation on continuous positive airway pressure (CPAP) efficiency in sleep apnoea/hypopnoea syndrome. Eur Respir J.

[B14] Teschler H, Berthon-Jones M (1998). Intelligent CPAP systems: clinical experience. Thorax.

[B15] Ayas NT, Patel SR, Malhotra A, Schulzer M, Malhotra M, Jung D, Fleetham J, White DP (2004). Auto-titrating versus standard continuous positive airway pressure for the treatment of obstructive sleep apnea: results of a meta-analysis. Sleep.

[B16] Fleetham J, Ayas N, Bradley D, Ferguson K, Fitzpatrick M, George C, Hanly P, Hill F, Kimoff J, Kryger M, Morrison D, Series F, Tsai W, CTS Sleep Disordered Breathing Committee (2006). Canadian Thoracic Society guidelines: diagnosis and treatment of sleep disordered breathing in adults. Can Respir J.

[B17] Sériès F, Marc I, Cormier Y, La Forge J (1994). Changes in the required levels of nasal continuous positive airway pressure in the course of treatment of obstructive sleep apnea. Eur Respir J.

[B18] Meurice JC, Marc I, Sériès F (1996). Efficiency of auto-CPAP in the treatment of obstructive sleep apnea/hypopnea syndrome. Am J Respir Crit Care Med.

[B19] Hudgel DW, Fung C (2000). A long-term randomized, cross-over comparison of auto-titrating and standard nasal continuous airway pressure. Sleep.

[B20] Randerath WJ, Schraeder O, Galetke W, Feldmeyer F, Ruhle KH (2001). Autoadjusting CPAP therapy based on impedance efficacy, compliance and acceptance. Am J Respir Crit Care Med.

[B21] Lloberes P, Ballester E, Montserrat JM, Botifoll E, Ramirez A, Reolid A, Gistau C, Rodriguez-Roisin R (1996). Comparison of manual and automatic CPAP titration in patients with sleep apnea/hypopnea syndrome. Am J Respir Crit Care Med.

[B22] Konerman M, Sanner BM, Vyleta M, Laschewiski F, Groetz J, Sturm A, Zideck W (1998). Use of conventional and self-adjusting nasal continuous positive airway pressure for treatment of severe obstructive sleep apnea syndrome. Chest.

[B23] Lofaso F, Lorino AM, Duizabo D, Najafi Zadeh H, Theret D, Goldenberg F, Harf A (1996). Evaluation of an auto-nCPAP device based on snoring detection. Eur Respir J.

[B24] Badia JR, Farre RO, John Kimoff R, Ballester E, Hernández L, Rotger M, Navajas D, Montserrat JM (1999). Clinical application of the forced oscillation technique for CPAP titration in the sleep apnea/hypopnea syndrome. Am J Respir Crit Care Med.

[B25] Farre R, Montserrat JM, Rigau J, Trepat X, Pinto P, Navajas D (2002). Response of automatic continuous positive airway pressure devices to different sleep breathing patterns: a bench study. Am J Respir Crit Care Med.

[B26] Abdenbi F, Chambille B, Escourrou P (2004). Bench testing of auto-adjusting positive airway pressure devices. Eur Respir J.

[B27] Stammnitz A, Jerrentrup A, Penzel T, Peter JH, Vogelmeier C, Becker HF (2004). Automatic CPAP titration with different self-setting devices in patients with obstructive sleep apnoea. Eur Respir J.

[B28] Pevernagie DA, Proot PM, Hertegonne KB, Neyens MC, Hoornaert KP, Pauwels RA (2004). Efficacy of flow- vs impedance-guided autoadjustable continuous positive airway pressure: a randomized cross-over trial. Chest.

[B29] Kessler R, Weitzenblum E, Chaouat A, Iamandi C, Alliotte T (2003). Evaluation of unattend automated titration to determine therapeutic continuous positive airway pressure in patients with obstructive sleep apnea. Chest.

[B30] Nolan GM, Ryan S, O'Connor TM, McNicholas WT (2006). Comparison of three auto-adjusting positive pressure devices in patients with sleep apnoea. Eur Respir J.

[B31] Vázquez JC, Tsai WH, Flemons WW, Masuda A, Brant R, Hajduk E, Whitelaw WA, Remmers JE (2000). Automated analysis of digital oximetry in the diagnosis of obstructive sleep apnoea. Thorax.

[B32] American Academy of Sleep Medecine Task Force Report (1999). Sleep-related breathing disorders in adults: Recommendations for syndrome definition and measurement techniques in clinical research. Sleep.

[B33] Sériès F, Kimoff RJ, Morrison D, Leblanc MH, Smilovitch M, Howlett J, Logan AG, Floras JS, Bradley TD (2005). Prospective evaluation of nocturnal oximetry for detection of sleep-related breathing disturbances in patients with chronic heart failure. Chest.

[B34] Mulgrew AT, Fox N, Ayas NT, Ryan CF (2007). Diagnosis and initial management of obstructive sleep apnea without polysomnography: a randomized validation study. Ann Intern Med.

[B35] West SD, Jones DR, Stradling JR (2006). Comparison of three ways to determine and deliver pressure during nasal CPAP therapy for obstructive sleep apnoea. Thorax.

[B36] Ayappa I, Norman RG, Hosselet JJ, Gruenke RA, Walsleben JA, Rapoport DM (1998). Relative occurrence of flow limitation and snoring during continuous positive airway pressure titration. Chest.

[B37] Haniffa M, Lasserson TJ, Smith I (2004). Interventions to improve compliance with continuous positive airway pressure for obstructive sleep apnoea. The Cochrane database of systematic reviews.

